# Pneumococcal Carriage Among Children and Adults Before and After a Change from a Three-dose 13-valent Pneumococcal Conjugate Vaccine Schedule without a Booster to a Two-Dose Schedule with a Booster in Burkina Faso

**DOI:** 10.1016/j.vaccine.2025.128064

**Published:** 2025-12-04

**Authors:** Lana Childs, Robert Lamoussa Zoma, Issa Ouedraogo, Guetwendé Sawadogo, T. Félix Tarbangdo, Aristide Zoma, Soumeya Ouangraoua, Emilie Dama, Theresa Tran, Fahmina Akhter, Simran Utreja, Mahamoudou Ouattara, Jennifer R. Verani, Soufiane Sanou, H. Flavien Aké, Lesley McGee, Miwako Kobayashi

**Affiliations:** 1 Centers for Disease Control and Prevention, Atlanta, GA, USA; 2 Davycas International, Ouagadougou, Burkina Faso; 3 Ministère de la Santé et de l’Hygiène Publique, Ouagadougou, Burkina Faso; 4 Centre Muraz, Bobo-Dioulasso, Burkina Faso; 5 Centers for Disease Control and Prevention, Ouagadougou, Burkina Faso; 6 CDC Foundation, Atlanta GA, USA

**Keywords:** Burkina Faso, pneumococcal vaccines, *Streptococcus pneumoniae*, carriage, administration & dosage, vaccine impact, pneumococcal conjugate vaccines

## Abstract

**Background:**

In June 2021, Burkina Faso changed the 13-valent pneumococcal conjugate vaccine (PCV13) schedule from three primary doses with no booster (at 2, 3, 4 months) (3+0) to two primary doses with a booster (at 2, 4, 9 months) (2+1), which may optimize vaccine impact due to a booster dose.

**Methods:**

We conducted two cross-sectional, age-stratified (age 1–11 months [infants], 1 year, 2–4 years, 5–14 years, and ≥15 years) pneumococcal carriage surveys in Bobo-Dioulasso in April–May 2022 and March–April 2023 and compared results with one similar survey conducted in March 2020. We collected demographic and epidemiologic information, including vaccination history (participants aged <5 years), and one nasopharyngeal (all participants) and one oropharyngeal (participants aged ≥5 years) swab from participants. Pneumococci isolated by culture were serotyped by PCR (all isolates in 2020 and 2022) and/or Quellung (subset of isolates in 2020 and 2022, all isolates in 2023). We evaluated pneumococcal carriage before (2020), during transition (2022), and after (2023) the PCV13 schedule change.

**Results:**

We enrolled 1,002, 1,025, and 1,007 participants in 2020, 2022, and 2023, respectively. Median age at dose 3 for 2+1-eligible children (infants in 2022 and children aged <2 years in 2023) was 4.3 months in 2022 and 9.1 months in 2023. From 2020 to 2023, PCV13-type carriage declined significantly among infants (2023: 9.5% 2020: 19.0%; adjusted prevalence ratio: 0.53, 95% CI: 0.31–0.88) but not in other age groups.

**Conclusions:**

The decline in PCV13-type carriage observed only in infants is likely unrelated to the PCV13 schedule change, since only those aged ≥9 months could have received a booster dose. Additional birth cohorts vaccinated with the 2+1 schedule are needed to understand the effects on PCV13-type carriage.

## Background

*Streptococcus pneumoniae* (pneumococcus) is a major cause of bacterial disease and associated deaths worldwide [1]. In 2021, the highest proportion of lower respiratory infections were due to *S. pneumoniae*, with an estimated 97.9 million episodes and 505,000 deaths globally [1]. In the African meningitis belt, pneumococcal meningitis has become a predominant cause of bacterial meningitis in many countries [2]. Pneumococci colonize the nasopharynx; while pneumococcal colonization is a precursor to disease, colonized individuals can transmit the bacteria person-to-person without developing disease [3]. Community pneumococcal carriage surveys evaluate the prevalence of colonization and can be an efficient way to assess changes in circulating serotypes and impact of pneumococcal conjugate vaccines (PCVs).

Introduction of PCVs led to marked declines in the incidence of disease caused by serotypes contained in the vaccines [4]. PCVs not only provide direct protection among vaccinated individuals but also provide indirect protection to unvaccinated populations through decreased transmission of vaccine serotypes in the community [4, 5]. The World Health Organization (WHO) recommends that countries introduce a PCV into the routine childhood immunization program using a schedule of either three primary doses without a booster (3+0) or two primary doses with a booster (2+1) [6]. Most countries in Africa introduced PCVs using a 3+0 schedule [7], and many sub-Saharan countries observed initial declines in vaccine-type (VT) carriage within 1–2 years after introduction followed by a stabilization at lower levels with continued transmission [8–10]. Several countries in the meningitis belt found persistent serotype 1 pneumococcal meningitis in the years following PCV introduction [2]. A PCV schedule with a booster dose may provide a longer duration of protection in vaccinated children and contribute to more pronounced indirect effects [6].

Burkina Faso is a country located in West Africa and entirely within the meningitis belt with a high burden of pneumococcal meningitis [11]. Burkina Faso introduced 13-valent PCV (PCV13) in October 2013 using a 3+0 schedule with doses administered at two, three, and four months of age, and third dose coverage was >90% for most years after introduction [12]. In the years following PCV13 introduction, VT disease declined, though serotype 1 (contained in PCV13) remained the predominant cause of pneumococcal meningitis after vaccine introduction [11]. Additionally, while VT carriage among colonized children aged <5 years declined by 41% from 2008 (the only pre-PCV13 data) and 2020 [13], persistent VT carriage was found approximately six years after PCV13 introduction in children (16–20%) and adults (6%) [8]. In June 2021, Burkina Faso changed the PCV13 schedule from 3+0 to 2+1 with doses administered at two, four, and nine months of age, making it the first country in the African meningitis belt to make the switch.

We conducted two cross-sectional, age-stratified community pneumococcal carriage surveys among children and adults after the PCV13 schedule change and compared results to a survey conducted before the schedule change [8] to assess the impact of the schedule change.

## Methods

### Study site and population

The surveys were conducted during April–May 2022 and March–April 2023 using the same methods and maintaining the same seasonality as the pre-schedule change survey conducted in March 2020 [8]. The surveys took place in Bobo-Dioulasso, the second largest city in Burkina Faso with an estimated population of 1.1 million in 2023 [14].

To recruit participants, we randomly selected 10 sectors with two back-ups among the 21 residential sectors in Bobo-Dioulasso (military and industrial sectors excluded). In each selected sector, all intersections were mapped, and 20 intersections were randomly selected with 10 back-ups. At each intersection, trained surveyors randomly selected a street by spinning a pen, and households on the selected street were visited consecutively starting on the left. Surveyors visited multiple households in the same intersection until one participant in each of five age groups was recruited: 1–11 months, 1 year, 2–4 years, 5–14 years, and ≥15 years. The sample size strategy was based on feasibility and budget; therefore, the same sample size was used in these surveys as prior surveys conducted in Bobo-Dioulasso after PCV13 introduction [8, 13]. Consistent with the 2020 survey, the target sample size in each survey was 1,000 participants or 200 per age group [8]. In households with multiple members in the same age group, surveyors used a simple random draw method to select one participant; however, multiple household members in different age groups were eligible for recruitment. The inclusion criteria were residents of Bobo-Dioulasso aged ≥1 month. The exclusion criteria were self-reported severe acute malnutrition or underlying disease.

### Data and specimen collection

At the household, written informed consent was obtained from adult participants or the parent/guardian of participants aged <18 years. The Burkina Faso Ethics Committee for Health Research does not require written informed assent from participants aged <18 years; however, the study background and procedures were explained to minor participants in age-appropriate language. Surveyors completed a questionnaire on demographic and epidemiologic characteristics of the household and participant. Vaccination history was collected from vaccination cards for children aged <5 years; if vaccination cards were missing during enrollment, field teams reviewed the immunization registers in local health posts. Lastly, participants were given appointments within 1–2 days of recruitment for clinical specimen collection at one of two district hospitals.

At the district hospitals, adult participants or parents/guardians of participants aged <18 years provided written informed consent for clinical specimen collection using the same consent forms as those used at the household. If a participant did not come to his or her clinic appointment, field supervisors made three attempts to follow up with the participant; after three attempts, the participant was replaced by another individual in the same age group living in the same intersection. Trained nurses completed a questionnaire on the recent health history of participants and collected nasopharyngeal swabs from all participants and oropharyngeal swabs from participants aged ≥5 years [15]. Nasopharyngeal and oropharyngeal swabs were immediately put into cryotubes containing 1 mL skim milk, tryptone, glucose, and glycerol (STGG) transport medium, and cryotubes were placed into coolers with ice packs. The coolers containing the cryotubes were transported to Centre Muraz, a national reference laboratory in Bobo-Dioulasso, within 4–6 hours of specimen collection.

### Laboratory methods

All nasopharyngeal and oropharyngeal swabs collected in 2020 and 2022 were analyzed at Centre Muraz. Upon arrival at Centre Muraz, laboratory technicians vortexed the inoculated STGG media for 10–20 seconds before placing the cryotubes in a −80°C freezer for storage. For swab analysis, 200μl of the swab-inoculated STGG media was transferred to 5.0 mL Todd Hewitt broth containing 0.5% yeast extract (THY) and supplemented with 1 mL of rabbit serum. This mixture was then incubated at 35–37°C for six hours. Following incubation, the cultured broth was streaked onto sheep blood agar plates for isolation and incubated in a 5% CO_2_ atmosphere at 35–37°C. After an incubation period of 18–24 hours, the plates were examined for the presence of alpha-hemolytic colonies. Pneumococci were identified based on their susceptibility to optochin and through bile solubility testing. All isolates of *S. pneumoniae* were inoculated into preservation medium (STGG) and stored at −80°C. The published sequential multiplex polymerase chain reaction (PCR) assay [16] was employed by Centre Muraz to determine pneumococcal serotypes. A subset comprising 20% of isolates serotyped by Centre Muraz, along with pneumococcal isolates with unresolved serotype results or those determined to be nontypeable (NT) by multiplex PCR, as well as all samples that tested negative, were sent to the Centers for Disease Control and Prevention (CDC) *Streptococcus* Laboratory in Atlanta, United States for quality control testing and serotyping by Quellung.

For the 2023 study, all swabs collected were initially stored at Centre Muraz before being shipped to CDC for further analysis. Pneumococcal isolation was performed using the aforementioned methods, while serotyping was completed using the Quellung method.

### Data management

Demographic and epidemiologic data collected during recruitment and clinical specimen collection were entered into an electronic ODK Collect form [17]. Deidentified pictures of vaccination cards were stored in the database in case of errors with data entry. Laboratory results were entered into a Microsoft Excel spreadsheet.

### Statistical analysis

We performed descriptive analyses of participant characteristics by survey year and compared differences between survey years using chi-square tests. For participants with both nasopharyngeal and oropharyngeal swabs collected (participants aged ≥5 years), the participant was considered colonized if pneumococci were detected by culture from either sample. We calculated the prevalence of overall pneumococcal carriage and VT carriage with 95% confidence intervals (CI) by survey year and age group. To estimate the pneumococcal carriage prevalence of all age groups combined, we applied weights to the prevalence estimates from each age group based on the population age distribution from the 2019 census projections for Houet Province where Bobo-Dioulasso is located [14]. Serotypes contained in PCV13 (1, 3, 4, 5, 6A, 6B, 7F, 9V, 14, 18C, 19A, 19F, and 23F) were considered VT. All other serotypes excluding NTs were considered nonvaccine-types (NVT).

We used the 2020 survey as the pre-schedule change baseline and the 2023 survey as the post-schedule change period and were the primary focus of this analysis. The 2022 survey was conducted to assess changes in pneumococcal carriage prevalence during COVID-19 and was considered a transition period to the 2+1 schedule as infants born in June 2021 (date of schedule change) started receiving their 9-month booster dose in January 2022 (approximately 3–4 months before the survey). Children aged <7 years in 2020, aged <9 years in 2022, and aged <10 years in 2023 were age-eligible for PCV13 since introduction. Children age-eligible for the 2+1 schedule were children aged <1 year in 2022 (though children aged <9 months were not eligible for the booster dose), and children aged <1 year and 1 year in 2023. All other children were eligible for the 3+0 schedule. We calculated crude and adjusted prevalence ratios (aPR) comparing VT pneumococcal colonization in the pre- (2020) and post-PCV13 (2023) schedule change surveys. We used standard methods to calculate crude prevalence ratios and log-binomial regression to estimate aPR; Poisson regression using robust error variance was used for aPR if log-binomial models failed to converge [18, 19]. To identify potential confounders, we assessed changes between surveys in the overall carriage prevalence by demographic and epidemiological characteristics. Those with statistically significant changes (*p* values <0.05) from 2020 to 2023 (presence of children aged <5 years in the household, ≥3 people sharing a bedroom) were included in aPRs. Age in years was included for the aPR for children aged 5–14 years and participants aged ≥15 years due to the wider age range of participants in these age groups. Prevalence ratios of all ages combined were weighted to account for the stratified sampling scheme. The carriage prevalence of individual serotypes between the pre- and post-PCV13 schedule change periods was compared using chi-square and Fisher’s exact tests for children aged <5 years combined, children aged 5–14 years, and participants aged ≥15 years. Data were analyzed using SAS software version 9.4. *P* values <0.05 or nonoverlapping 95% CIs between two carriage prevalence estimates were considered statistically significant.

### Ethical considerations

The Burkina Faso Ethics Committee for Health Research approved the study protocols (N°2022–04–071 and N°2023–01–008). This activity was reviewed by CDC, deemed not research, and was conducted consistent with applicable federal law and CDC policy^§^.

## Results

### Household and participant characteristics

We enrolled 1,002 participants in 2020, 1,025 participants in 2022, and 1,007 participants in 2023 (Table 1). Within the 1–11-month-old age group, 76.0% and 68.2% were aged <9 months at the time of enrollment in 2022 and 2023, respectively, and therefore, too young to have received a booster dose in the 2+1 schedule. In 2022 and 2023 compared to 2020, there were significantly fewer households with ≥3 persons sharing a bedroom (2020: 54.8%, 2022: 43.8%, 2023: 45.4%, 2022 vs. 2020 *p*<0.0001, 2023 vs. 2020 *p*<0.0001), a motorbike as household possession (2020: 86.1%, 2022: 81.3%, 2023: 74.6%, 2022 vs. 2020 *p*=0.003, 2023 vs. 2020 *p*<0.0001), and participants self-reporting recent antibiotic use (2020: 12.9%, 2022: 8.6%, 2023: 7.2%, 2022 vs. 2020 *p*=0.002, 2023 vs. 2020 *p*<0.0001). Declines were also found among participants reporting a recent acute respiratory illness, though changes were only significant from 2020 to 2022 (2020: 60.7%, 2022: 43.5%, 2022 vs. 2020 *p*<0.0001). From 2020 to 2023, the presence of other children aged <5 years in the household (2020: 88.7%, 2023: 93.7%, 2023 vs. 2020 *p*<0.0001) and households possessing a radio (2020: 71.6%, 2023: 90.8%, 2023 vs. 2020 *p*<0.0001) increased significantly, though there was a decline in radio possession in 2022 (2022: 58.3%, 2022 vs. 2020 *p*<0.0001). All other household and participant characteristics remained similar across the survey years.

### PCV13 vaccination history

**Figure 1.**
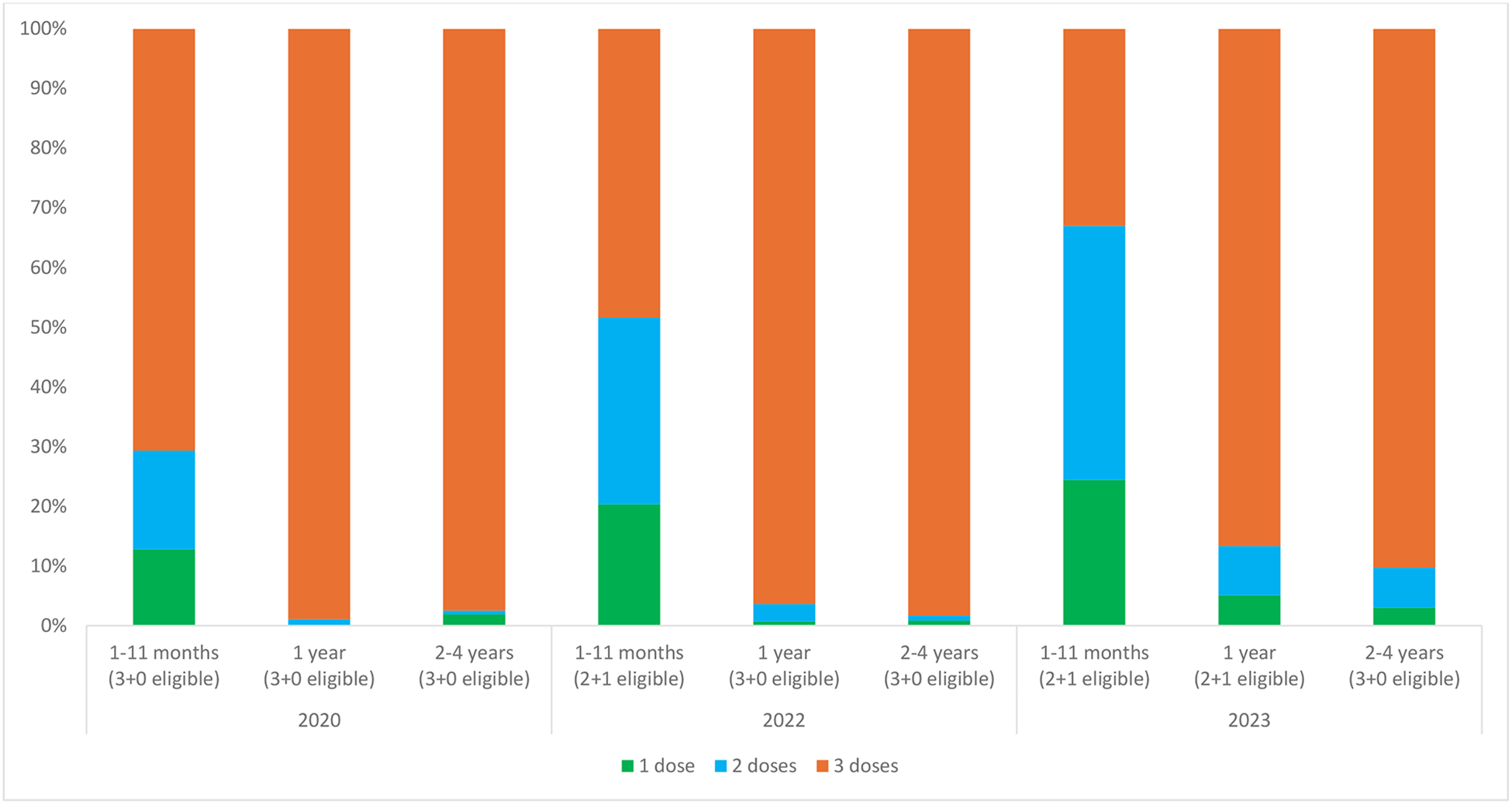
Number of PCV13 Doses Among Children Aged <5 Years with Card-Confirmed Vaccination History by Survey Year, Age Group, and PCV13 Schedule Eligibility^a^, Bobo-Dioulasso, Burkina Faso PCV13: 13-valent pneumococcal conjugate vaccine ^a^Children age-eligible for the 2+1 schedule were children aged <1 year in 2022 (though children aged <9 months were not eligible for the booster dose), and children aged <1 year and 1 year in 2023. All other children were eligible for the 3+0 schedule.

PCV13 vaccination history was available by card/registry or verbal report for 90.0%, 74.8%, and 93.5% of children aged <5 years in 2020, 2022, and 2023, respectively (Table 2). Among those children, prior receipt of PCV13 was confirmed for most by card or registry (2020: 92.8%, 2022: 83.0%, 2023: 95.2%). In 2020, all children aged <5 years were eligible for the 3+0 schedule, and among those with card or registry confirmed PCV13 history, the proportion who received 3 doses ranged from 70.7% (children aged 1–11 months) to 98.9% (children aged 1 year), while the median age range at dose 3 was 4.2 months for both age groups aged <2 years and 4.4 months for children aged 2–4 years (Figure 1, Table 2). In 2022, among 2+1 eligible children (aged 1–11 months) with card or registry confirmed PCV13 history, 48.4% had received 3 doses and the median age at dose 3 was similar to that of 2020 at 4.3 months (interquartile range [IQR]: 4.1, 4.8). In 2023, the median age at dose 3 among 2+1 eligible children (aged <2 years) had shifted to 9.1 months among children aged 1–11 months (IQR: 4.4, 9.3) and 1 year (IQR: 5.3, 9.7). In 2023, the proportion of children with card or registry confirmed PCV13 who received 3 doses was 33.0% among children aged 1–11 months, 86.7% among children aged 1 year, and 90.3% among children aged 2–4 years (3+0 eligible).

### Changes in overall pneumococcal carriage prevalence

Among participants of all ages combined, overall carriage prevalence remained stable in the pre- and post-PCV13 schedule change surveys (2020: 40.9%, 95% CI: 37.0–44.8; 2023: 41.4%, 95% CI: 37.2–45.5) (Table 3). By age group, no significant changes were found from 2020 to 2023, except among children aged 2–4 years (2020: 73.1%, 95% CI: 67.0–79.3 vs. 2023: 58.0%, 95% CI: 51.1–64.9). Notably, in 2022, the overall carriage prevalence among participants of all ages combined and by age group was significantly lower than in 2020 (except participants aged ≥15 years) and 2023.

### Changes in VT carriage prevalence

Pre- and post-schedule change, there were no significant changes in VT carriage among all pneumococcal carriers (2020: 26.4%, 95% CI: 21.2–31.7 vs. 2023: 34.1%, 95% CI: 28.1–40.1) or by age group (Table 3) although there was a non-significant decline among children aged 1–11 months (27.3% in 2020 to 15.1% in 2023), unlike other age groups.

**Figure 2.**
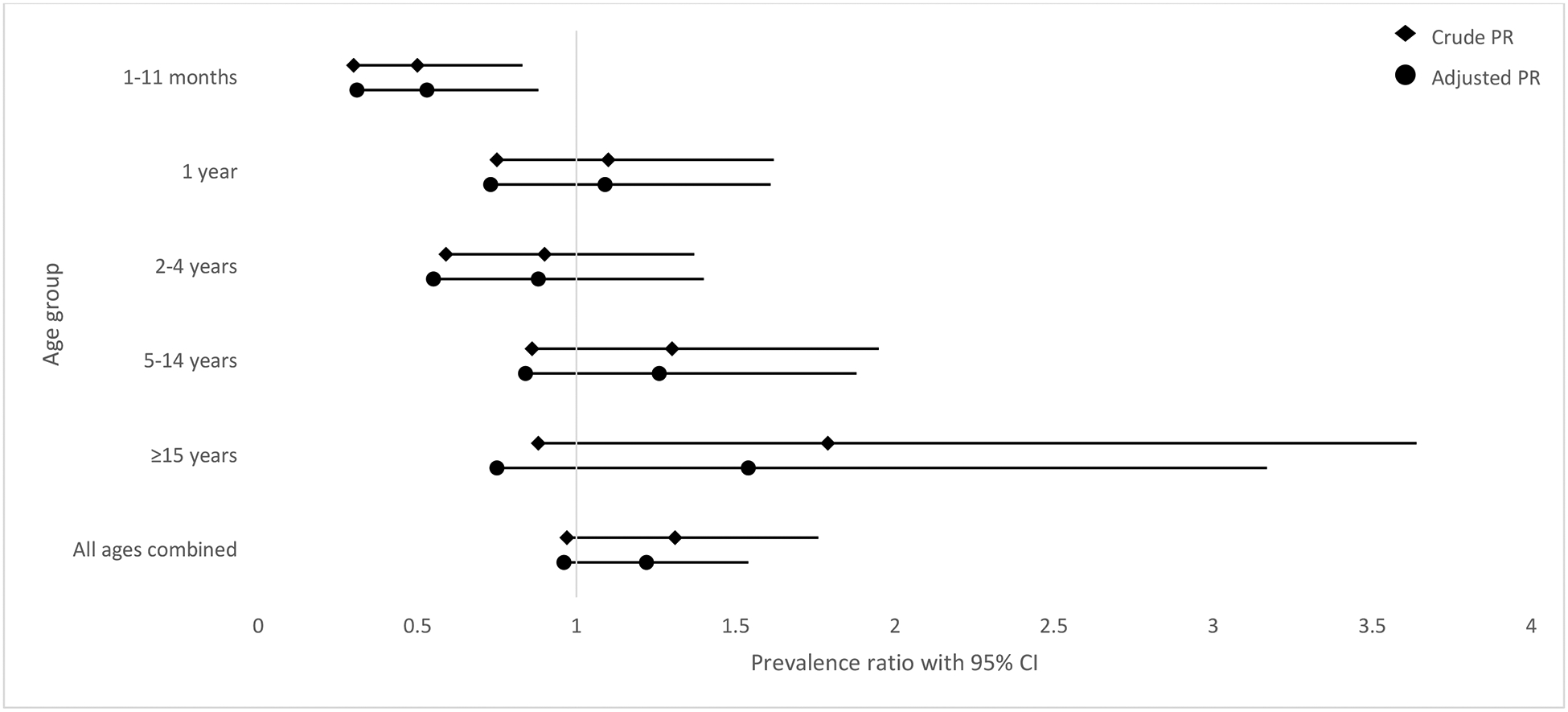
Crude and Adjusted^a^ Prevalence Ratios for Vaccine-type Carriage^b^ Pre- (2020) and Post- (2023) PCV13 schedule Change, Bobo-Dioulasso, Burkina Faso CI: confidence interval; PR: prevalence ratio ^a^Adjusted for presence of children aged <5 years in the household and ≥3 people sharing a bedroom. The aPR for children aged 5–14 years and participants aged ≥15 years were also adjusted for age in years. ^b^Vaccine-type carriage is defined as carriage with serotypes included in 13-valent pneumococcal conjugate vaccine (1, 3, 4, 5, 6A, 6B, 7F, 9V, 14, 18C, 19A, and 23F).

There were no statistically significant changes in VT carriage prevalence among all ages (2020: 10.8% vs. 2023: 14.1%; aPR: 1.22, 95% CI: 0.96–1.54) (Table 3, Figure 2). By age group, VT carriage declined significantly only among children aged 1–11 months (2020: 19.0% vs. 2023: 9.5%; aPR: 0.53, 95% CI: 0.31–0.88).

### Changes in serotype-specific carriage prevalence

**Figure 3.**
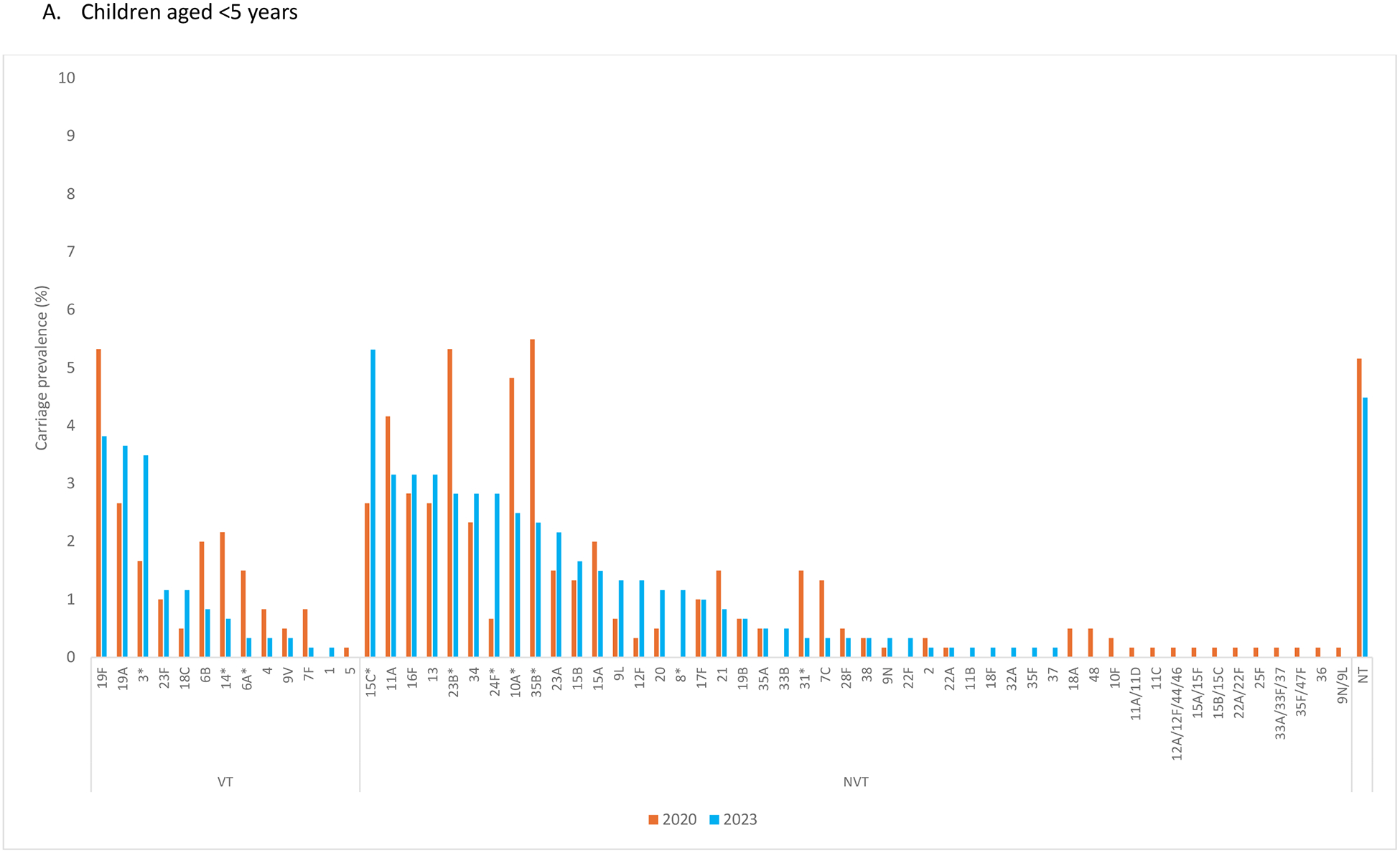

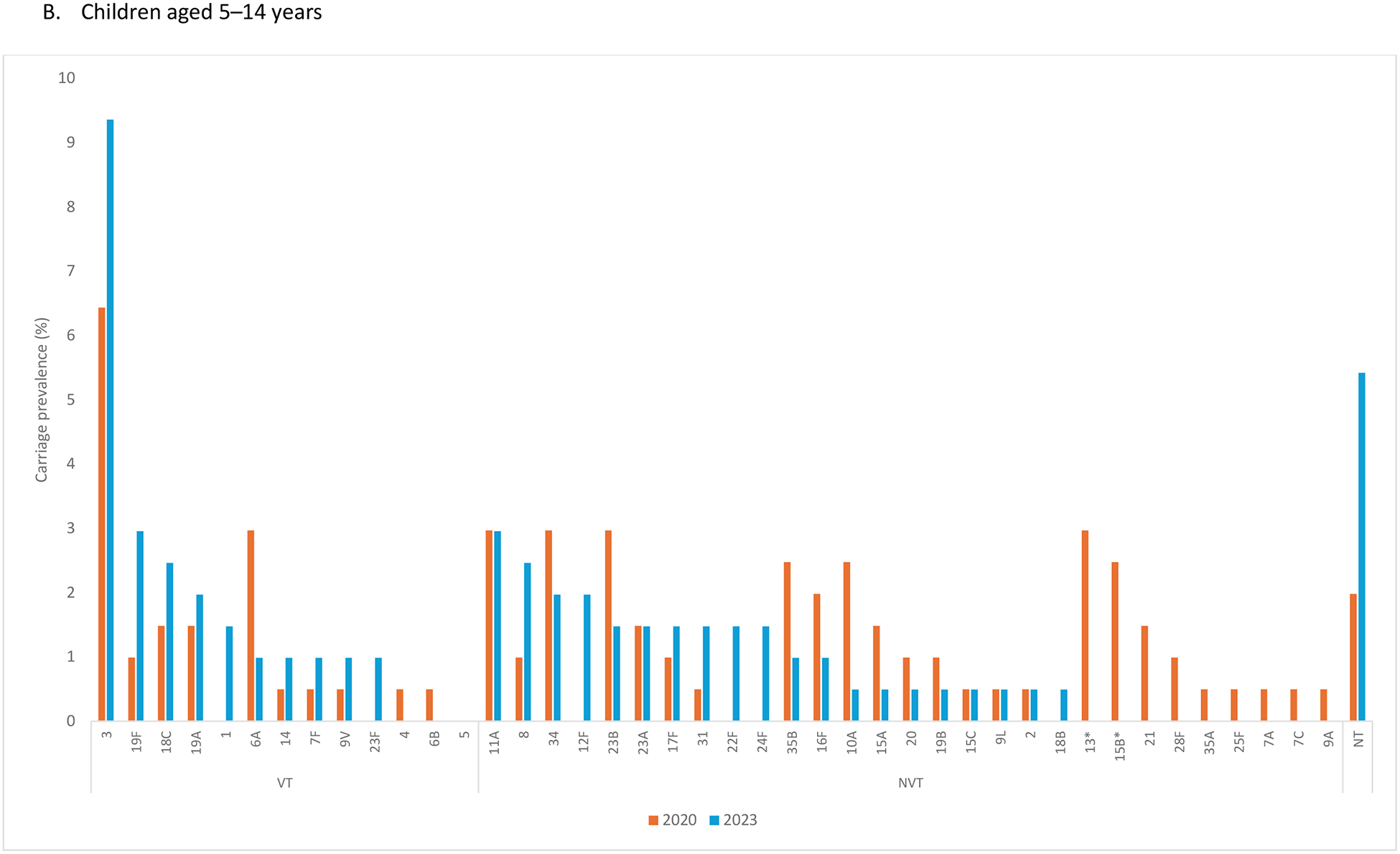

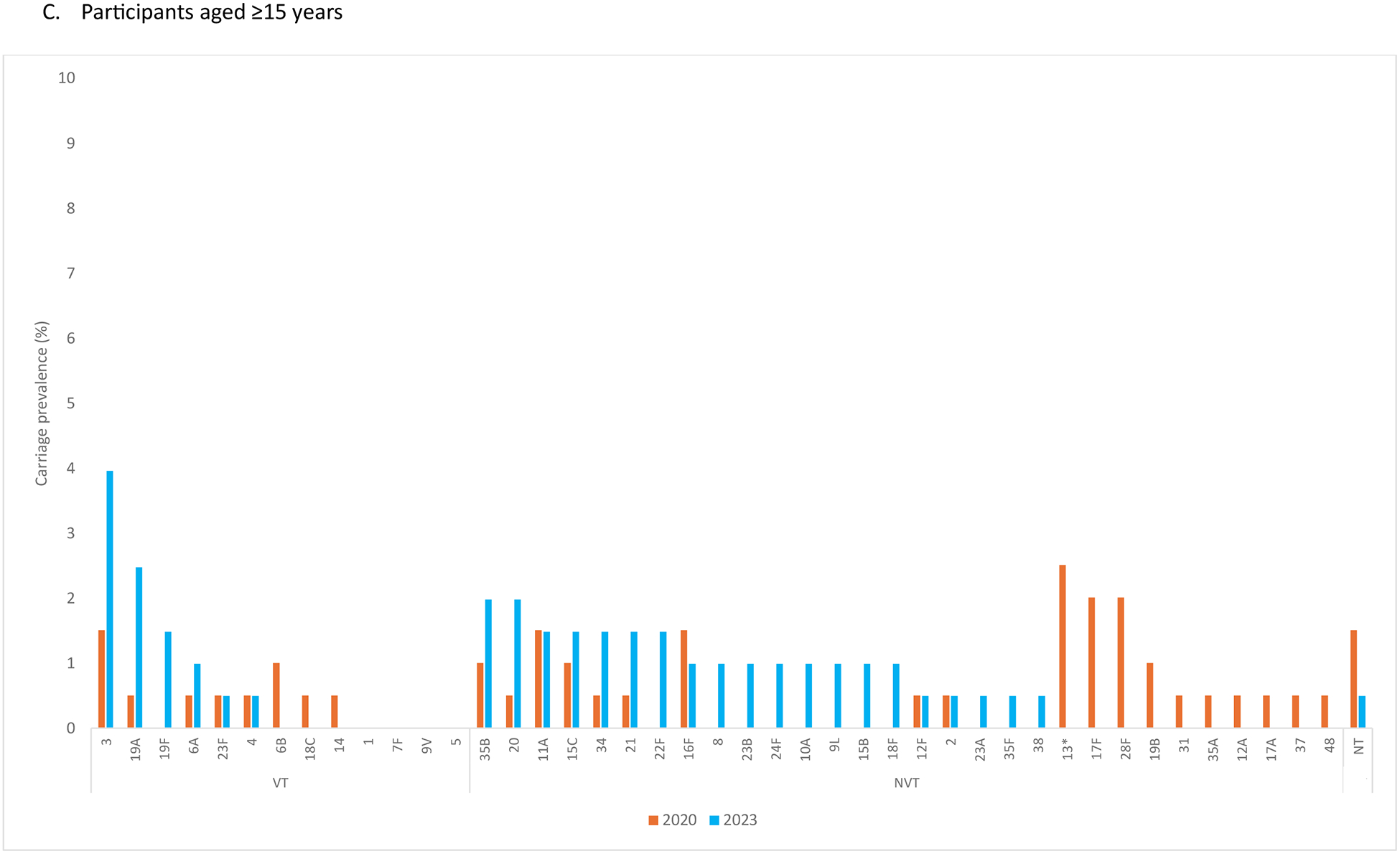
Serotype-specific pneumococcal carriage prevalence in 2020 and 2023, Bobo-Dioulasso, Burkina Faso Abbreviations: NT: nontypeable; NVT: nonvaccine-type; VT: vaccine-type *p-value <0.05

Among children aged <5 years, serotypes 19F (3.8%, 23/602), 19A (3.7%, 22/602), and 3 (3.5%, 21/602) were the most common VT and serotypes 15C, 11A, and 16F were the most common NVT in 2023 (Figure 3A). From 2020 to 2023, carriage prevalence increased significantly for serotypes 3 (VT) (1.7% vs. 3.5%), 15C (NVT) (2.7% vs. 5.3%), 24F (NVT) (0.7% vs. 2.8%), and 8 (NVT) (0.0% vs. 1.2%) and decreased significantly for serotypes 14 (VT) (2.2% vs. 0.7%), 6A (VT) (1.5% vs. 0.3%), 23B (NVT) (5.3% vs. 2.8%), 10A (NVT) (4.8% vs. 2.5%), 35B (NVT) (5.5% vs. 2.3%), and 31 (NVT) (1.5% vs. 0.3%) among children aged <5 years. Among children aged 1–11 months, serotypes 19A (2.0%, 4/201), 3 (1.5%, 3/201) and 19F (1.0%, 2/201) were the most common VT in 2023, and from 2020 to 2023, declines were noted across multiple VT, with the largest reduction in serotype 19F (6.0% vs. 1.0%). Among children aged 5–14 years and participants aged ≥15 years, serotype 3 had the highest carriage prevalence among VT in 2023, while the most common NVT varied between these two age groups (Figure 3B and 3C).

## Discussion

In Burkina Faso, a setting where VT carriage persisted in children and adults after six years of PCV13 use despite high vaccination coverage [8, 12], we found significant reductions in the prevalence of VT carriage among children aged 1–11 months from pre- to post-schedule change. However, in the post-schedule change survey conducted <2 years after the schedule change and approximately one year after infants started receiving their booster doses, we found no evidence of indirect effects of the schedule change on older age groups. Additionally, approximately one in five children aged 1–14 years and one in 10 participants aged ≥15 years remained colonized with a VT.

Overall carriage prevalence generally remains stable over time after PCV introduction, as VT declines and NVT increases in circulation [20]. Therefore, we would not expect a change in PCV schedule to impact overall carriage prevalence. From 2020 to 2022, we found significant declines in the overall carriage prevalence among all ages combined and for all age groups (largest decline was in children aged 2–4 years); by the 2023 survey, the overall carriage prevalence for all ages combined and for most age groups (except children aged 2–4 years) had increased back to prevalences seen in 2020. It is possible that pneumococcal transmission was impacted by COVID-19 mitigation measures [21, 22], leading to a lower carriage prevalence in 2022, and that easing of those measures allowed pneumococcal carriage to return to pre-pandemic levels by 2023. Some studies reported stable pneumococcal carriage during the pandemic [23, 24], while others reported a decline in carriage followed by a return to levels seen pre-pandemic [21, 22]. Findings from our surveys suggest that in a high-transmission setting, COVID-19 mitigation measures may result in a transient decline in overall carriage prevalence.

From 2020 to 2023, VT carriage prevalence declined in children aged 1–11 months, remained stable in children aged 1 year and 2–4 years, and increased (non-significantly) in older children and participants aged ≥15 years. Among children aged 1–11 months, there was a small non-significant decline in the prevalence of overall carriage from 2020 to 2023; however, the significant reduction in VT carriage in this age group was primarily driven by reductions in VT carriage among pneumococcal carriers (2020: 27.3%, 2023: 15.1%), suggesting a vaccine-related effect among infants pre- and post-schedule change. Yet most infants in 2023 (i.e., all those aged <9 months) were too young to have received a booster dose. In fact, this age group may face a slight increased risk of VT carriage acquisition between completion of the two primary dose series and the 9-month booster dose compared to receiving all three doses as a primary series earlier in infancy. We explored other possible reasons for the significant decline in VT carriage, including significant changes in the characteristics (e.g., recent antibiotic use or acute respiratory illness) of infants pre- and post-schedule change; however, none were found. We do not believe the decline in VT carriage in infants in 2023 is attributable to the schedule change, especially since no changes were observed among children aged 1 year, which is when the potential benefits of a booster-containing schedule would most likely be apparent.

The lack of evidence of indirect effects in older age groups may be because insufficient time had passed for indirect effects to accumulate. At the time of the 2023 survey, only two birth cohorts were eligible for the 2+1 schedule (some of which were not yet old enough to receive a booster dose), and our findings on the median age at dose 3 suggest that implementation of the new schedule was suboptimal in 2022. In many settings, including sub-Saharan Africa, indirect effects on VT carriage were observed within 1–3 years of PCV introduction [25–28]. However, in other settings, the indirect effects of PCV introduction took longer to accumulate and were not observed until 5–7 years of PCV introduction with high coverage [29, 30]. In Burkina Faso, evidence of indirect effects of PCV13 introduction on VT carriage was not seen until approximately six years after introduction [8]; thus, any indirect effects of the schedule change may similarly take several years to become apparent. Additionally, because the schedule change occurred nearly eight years after PCV13 introduction, the incremental change in VT carriage from the schedule change will likely be much smaller than the impact of PCV13 introduction in a vaccine-naïve population. Additional data collected after a longer period of implementation of the 2+1 schedule may be needed to understand the potential indirect effects.

In 2023, serotype 3 was the most common VT among children aged 5–14 years and participants aged ≥15 years, and we found significant increases in the carriage prevalence of serotype 3 among children aged <5 years. This finding is consistent with other PCV13-using countries in Africa that found serotype 3 to be one of the predominant VT in post-PCV introduction carriage studies [9, 10, 31], likely due to PCV13 being less immunogenic for serotype 3 compared to other VT [32]. In Burkina Faso, serotype 3 is not a common cause of pneumococcal meningitis [11]; however, additional monitoring of serotype 3 pneumococcal meningitis is warranted as serotype 3 requires higher IgG concentrations to prevent invasive pneumococcal disease (IPD) compared with other VT [33], and a recent study from South Africa found little to no reduction in serotype 3 IPD many years after PCV13 introduction [34].

This assessment is subject to several limitations. First, there may be unmeasured factors that resulted in the significant decline in overall carriage prevalence in 2022, and we cannot rule out residual effects of these factors in the 2023 survey. Second, the sample size was based on feasibility and budget, and we used the same sample sized as prior surveys conducted in Bobo-Dioulasso after PCV13 introduction [8, 13]; therefore, we may have been underpowered to detect significant declines, especially in participants aged ≥15 years as VT carriage prevalence is lowest in this age group. Third, this assessment did not account for clustering of participants in different age groups recruited from the same household due to the sampling method used in the surveys. Fourth, we used the provincial level population age distribution for the weight calculations as this was the best available estimate of the age distribution in the population; however, it may not be truly representative of the age distribution in Bobo-Dioulasso. Fifth, due to the limited timeframe between when infants started receiving their booster doses and the 2023 survey, it is possible that insufficient time had passed for indirect effects to accumulate. Lastly, in 2020 and 2022, the laboratory analysis was conducted by Centre Muraz in Bobo-Dioulasso, and a subset of samples and isolates were sent to CDC Atlanta for quality control testing, while in 2023, all specimens were sent to and subsequently analyzed at CDC Atlanta, which may have resulted in slight differences in pneumococcal isolation or serotyping.

## Conclusion

This assessment provides the first insights into pneumococcal carriage in Burkina Faso following the PCV13 schedule change. As the post-schedule change survey took place <2 years after the PCV13 schedule change, additional monitoring of VT transmission in the community is needed to understand the mid- and long-term impacts of the schedule change, especially given that in January 2025, the Ministère de la Santé et de l’Hygiène changed the PCV13 schedule for a second time, moving the 9-month booster to 15 months. To our knowledge, Burkina Faso is only one of two countries in sub-Saharan Africa to switch from a 3+0 to a 2+1 schedule, and the findings from this assessment will be important for other countries in Africa with a high pneumococcal disease burden considering a PCV schedule change.

## Figures and Tables

**Table 1.** Demographic and Epidemiological Characteristics of Enrolled Participants by Survey Year, Bobo-Dioulasso, Burkina Faso

	2020 N=1002		2022 N=1025		2023 N=1007		2022 vs. 2020 *P* value	2023 vs. 2020 *P* value
Characteristics	n	%	n	%	n	%		
Age group								
1–11 months	200	20.0	208	20.3	201	20.0	1.0	1.0
1 year	200	20.0	204	19.9	201	20.0		
2–4 years	201	20.1	209	20.4	200	19.9		
5–14 years	202	20.2	203	19.8	203	20.2		
≥15 years	199	19.9	201	19.6	202	20.1		
Female sex	548	54.7	583	56.9	536	53.2	0.322	0.511
Presence of other children aged <5 years in the household^a^	889	88.7	-	-	944	93.7	-	<0.0001
Household size								
1–4	307	30.6	342	33.4	283	28.1	0.154	0.271
5–6	353	35.2	373	36.4	348	34.6		
≥7	342	34.1	310	30.2	376	37.3		
≥3 persons sharing a bedroom	453	54.8	449	43.8	457	45.4	<0.0001	<0.0001
Children in household attending daycare or school	777	77.5	797	77.8	762	75.7	0.909	0.321
Cigarette smoker in household	152	15.2	163	15.9	148	14.7	0.649	0.766
Fuel source^b^								
Gas	115	11.5	197	19.2	95	9.4	-	0.009
Coal	709	70.8	709	69.2	773	76.8		
Wood	178	17.8	290	28.3	139	13.8		
Cooking location^c^								
Inside	455	45.4	184	18.0	149	14.8	-	-
Under semi-enclosed structure	141	14.1	136	13.3	208	20.7	-	-
Outside	662	66.1	705	68.8	650	64.6	-	-
Household possessions								
Radio	717	71.6	598	58.3	914	90.8	<0.0001	<0.0001
Television	885	88.3	901	87.9	877	87.1	0.770	0.400
Phone	996	99.4	1005	98.1	980	97.3	0.007	0.0002
Motorbike	863	86.1	833	81.3	751	74.6	0.003	<0.0001
Car	84	8.4	77	7.5	64	6.4	0.468	0.082
Acute respiratory illness in past two weeks^d^	608	60.7	446	43.5	571	56.7	<0.0001	0.070
Antibiotic use in the past two weeks	129	12.9	88	8.6	72	7.2	0.002	<0.0001

a Information on other children aged <5 years living in the household was not included in the 2022 questionnaire.

b Multiple responses possible in 2022 while only one response possible in 2020 and 2023.

c Multiple responses possible in 2020 while only one response possible in 2022 and 2023.

d Acute respiratory illness symptoms include runny nose, sore throat, cough, and/or fever.

**Table 2.** Routine PCV13 History Among Children Aged <5 Years by Survey Year, Bobo-Dioulasso, Burkina Faso

	2020		2022		2023	
	n	%	n	%	n	%
Availability of PCV13 vaccination history^a^	n=599		n=615		n=602	
Card or registry confirmed or verbal report	539	90.0	460	74.8	563	93.5
Unknown	60	10.0	155	25.2	39	6.5
Receipt of any PCV13 dose (among those with card or registry confirmed or verbal report)	n=539		N=460		n=563	
Card or registry confirmed	500	92.8	382	83.0	536	95.2
Verbal report	39	7.2	78	17.0	27	4.8
Median age with IQR at PCV13 dose 3 (among those with card or registry confirmed)^b^	n=443		n=296		N=366	
Children aged 1–11 months	116	4.2 (4.1, 4.6)	58	4.3 (4.1, 4.8)	56	9.1 (4.4, 9.3)
Children aged 1 year	176	4.2 (4.0, 4.8)	128	4.3 (4.1, 5.0)	166	9.1 (5.3, 9.7)
Children aged 2–4 years	151	4.4 (4.1, 5.1)	110	4.3 (4.1, 4.6)	144	4.4 (4.1, 5.1)

PCV13: 13-valent pneumococcal conjugate vaccine; IQR: interquartile range

a Denominator excludes missing data.

b Median age with IQR calculated for children with date of birth available and card or registry confirmed date of PCV13 dose 3.

**Table 3.** Pneumococcal Carriage Prevalence by Age Group and Survey Year, Bobo-Dioulasso, Burkina Faso

	2020		2022		2023	
	n/N	% (95% CI)	n/N	% (95% CI)	n/N	% (95% CI)
Overall pneumococcal carriage^a^	594/1002	40.9 (37.0–44.8)	362/1025	25.7 (22.1–29.3)	554/1007	41.4 (37.2–45.5)
1–11 months	139/200	69.5 (63.1–75.9)	89/208	42.8 (36.1–49.5)	126/201	62.7 (56.0–69.4)
1 year	151/200	75.5 (69.5–81.5)	102/204	50.0 (43.1–56.9)	149/201	74.1 (68.1–80.2)
2–4 years	147/201	73.1 (67.0–79.3)	68/209	32.5 (26.2–38.9)	116/200	58.0 (51.1–64.9)
5–14 years	107/202	53.0 (46.1–59.9)	65/203	32.0 (25.6–38.4)	99/203	48.8 (41.9–55.7)
≥15 years	50/199	25.1 (19.1–31.2)	38/201	18.9 (13.5–24.3)	64/202	31.7 (25.3–38.1)
VT carriage among pneumococcal carriers^a,b^	159/594	26.4 (21.2–31.7)	93/362	33.5 (25.8–41.2)	159/554	34.1 (28.1–40.1)
1–11 months	38/139	27.3 (19.9–34.8)	11/89	12.4 (5.5–19.2)	19/126	15.1 (8.8–21.3)
1 year	39/151	25.8 (18.8–32.8)	22/102	21.6 (13.5–29.6)	43/149	28.9 (21.6–36.2)
2–4 years	38/147	25.9 (18.8–32.9)	22/68	32.4 (21.2–43.5)	34/116	29.3 (21.0–37.6)
5–14 years	33/107	30.8 (22.1–39.6)	25/65	38.5 (26.5–50.3)	43/99	43.4 (33.6–53.2)
≥15 years	11/50	22.0 (10.5–33.5)	13/38	34.2 (19.1–49.4)	20/64	31.2 (19.9–42.6)
VT carriage among all ages^a,b^	159/1002	10.8 (8.5–13.2)	93/1025	8.6 (6.3–10.9)	159/1007	14.1 (11.3–17.0)
1–11 months	38/200	19.0 (13.6–24.4)	11/208	5.3 (2.2–8.3)	19/201	9.5 (5.4–13.5)
1 year	39/200	19.5 (14.0–25.0)	22/204	10.8 (6.5–15.0)	43/201	21.4 (15.7–27.1)
2–4 years	38/201	18.9 (13.5–24.3)	22/209	10.5 (6.4–14.7)	34/200	17.0 (11.8–22.2)
5–14 years	33/202	16.3 (11.2–21.4)	25/203	12.3 (7.8–16.8)	43/203	21.2 (15.6–26.8)
≥15 years	11/199	5.5 (2.3–8.7)	13/201	6.5 (3.1–9.9)	20/202	9.9 (5.8–14.0)

CI: confidence interval; VT: vaccine-type

a To estimate the pneumococcal carriage prevalence of all age groups combined, we applied weights to the prevalence estimates from each age group based on the age distribution of the population derived from the 2019 census population projections (14).

b VT carriage is defined as carriage with serotypes included in 13-valent pneumococcal conjugate vaccine (PCV13) (1, 3, 4, 5, 6A, 6B, 7F, 9V, 14, 18C, 19A, and 23F).

## Footnotes

§ See e.g., 45 C.F.R. part 46.102(I)(2), 21 C.F.R. part 56: 42 U.S.C §241(d); 5 U.S.C. §552a; 44 U.S.C. §3501 et seq.

## References

[1] Infections GBDLR, Antimicrobial Resistance C. Global, regional, and national incidence and mortality burden of non-COVID-19 lower respiratory infections and aetiologies, 1990–2021: a systematic analysis from the Global Burden of Disease Study 2021. Lancet Infect Dis. 2024;24:974–1002. 38636536 10.1016/S1473-3099(24)00176-2 PMC11339187

[2] Franklin K, Kwambana-Adams B, Lessa FC, Soeters HM, Cooper L, Coldiron ME, Pneumococcal Meningitis Outbreaks in Africa, 2000–2018: Systematic Literature Review and Meningitis Surveillance Database Analyses. J Infect Dis. 2021;224:S174–S83. 34469561 10.1093/infdis/jiab105 PMC8414910

[3] Simell B, Auranen K, Kayhty H, Goldblatt D, Dagan R, O’Brien KL, The fundamental link between pneumococcal carriage and disease. Expert Rev Vaccines. 2012;11:841–55. 22913260 10.1586/erv.12.53

[4] Bennett JC, Deloria Knoll M, Kagucia EW, Garcia Quesada M, Zeger SL, Hetrich MK, Global impact of ten-valent and 13-valent pneumococcal conjugate vaccines on invasive pneumococcal disease in all ages (the PSERENADE project): a global surveillance analysis. Lancet Infect Dis. 2024. 10.1016/S1473-3099(24)00665-0 PMC11947069 39706204

[5] Shiri T, Datta S, Madan J, Tsertsvadze A, Royle P, Keeling MJ, Indirect effects of childhood pneumococcal conjugate vaccination on invasive pneumococcal disease: a systematic review and meta-analysis. Lancet Glob Health. 2017;5:e51–e9. 27955789 10.1016/S2214-109X(16)30306-0

[6] World Health Organization. Pneumococcal conjugate vaccines in infants and children under 5 years of age: WHO position paper. Weekly epidemiological record. 2019;94:85–104.

[7] International Vaccine Access Center (IVAC) Johns Hopkins Bloomberg School of Public Health. www.view-hub.org. [March 26, 2025].

[8] Childs L, Ouedraogo I, Zoma RL, Tarbangdo TF, Sawadogo G, Ake HF, Pneumococcal Carriage in Burkina Faso After 13-Valent Pneumococcal Conjugate Vaccine Introduction and Before a Schedule Change. Open Forum Infect Dis. 2024;11:ofae303. 38911949 10.1093/ofid/ofae303 PMC11191361

[9] Usuf E, Bottomley C, Gladstone R, Bojang E, Jawneh K, Cox I, Persistent and Emerging Pneumococcal Carriage Serotypes in a Rural Gambian Community After 10 Years of Pneumococcal Conjugate Vaccine Pressure. Clin Infect Dis. 2021;73:e3825–e35. 32584973 10.1093/cid/ciaa856

[10] Swarthout TD, Fronterre C, Lourenco J, Obolski U, Gori A, Bar-Zeev N, High residual carriage of vaccine-serotype Streptococcus pneumoniae after introduction of pneumococcal conjugate vaccine in Malawi. Nat Commun. 2020;11:2222. 32376860 10.1038/s41467-020-15786-9 PMC7203201

[11] Soeters HM, Kambire D, Sawadogo G, Ouedraogo-Traore R, Bicaba B, Medah I, Impact of 13-Valent Pneumococcal Conjugate Vaccine on Pneumococcal Meningitis, Burkina Faso, 2016–2017. J Infect Dis. 2019;220:S253–S62. 31671444 10.1093/infdis/jiz301 PMC8935360

[12] World Health Organization. https://www.who.int/teams/immunization-vaccines-and-biologicals/immunization-analysis-and-insights/global-monitoring/immunization-coverage/who-unicef-estimates-of-national-immunization-coverage. [March 27, 2025].

[13] Kabore L, Adebanjo T, Njanpop-Lafourcade BM, Ouangraoua S, Tarbangdo FT, Meda B, Pneumococcal Carriage in Burkina Faso After 13-Valent Pneumococcal Conjugate Vaccine Introduction: Results From 2 Cross-sectional Population-Based Surveys. J Infect Dis. 2021;224:S258–S66. 34469552 10.1093/infdis/jiab037 PMC8409529

[14] Comité National du Recensement. Institut National de la Statistique et de la Démographie. https://www.insd.bf/fr/resultats. [April 2, 2025].

[15] Satzke C, Turner P, Virolainen-Julkunen A, Adrian PV, Antonio M, Hare KM, Standard method for detecting upper respiratory carriage of Streptococcus pneumoniae: updated recommendations from the World Health Organization Pneumococcal Carriage Working Group. Vaccine. 2013;32:165–79. 24331112 10.1016/j.vaccine.2013.08.062

[16] Centers for Disease Control and Prevention Streptococcus Laboratory. https://www.cdc.gov/streplab/pneumococcus/resources.html. [April 12, 2024].

[17] ODK. https://getodk.org/. [March 19, 2025].

[18] Zou G A modified poisson regression approach to prospective studies with binary data. Am J Epidemiol. 2004;159:702–6. 15033648 10.1093/aje/kwh090

[19] Spiegelman D, Hertzmark E. Easy SAS calculations for risk or prevalence ratios and differences. Am J Epidemiol. 2005;162:199–200. 15987728 10.1093/aje/kwi188

[20] Tvedskov ESF, Hovmand N, Benfield T, Tinggaard M. Pneumococcal carriage among children in low and lower-middle-income countries: A systematic review. Int J Infect Dis. 2022;115:1–7. 34800691 10.1016/j.ijid.2021.11.021

[21] Fuji N, Gonzalez E, Salamone FN, Bajorski P, Kaur R, Pichichero M. Comparison of Streptococcus pneumoniae nasopharyngeal colonization, serotype-specific and protein-specific antibody and cytokine levels in young children prior to, during and post COVID-19 pandemic. Vaccine. 2025;54:126954. 40058284 10.1016/j.vaccine.2025.126954

[22] Charnogursky CE, Gil AI, Ecker L, Cornejo R, Rios S, Ochoa M, Pandemic Social Distancing and Declines in Nasopharyngeal Carriage of Pneumococcus and Related Antimicrobial-Resistant Genes: Evidence From Household-Based Cohort Studies in Lima, Peru. Open Forum Infect Dis. 2025;12:ofaf157. 40242076 10.1093/ofid/ofaf157 PMC12000526

[23] Willen L, Ekinci E, Cuypers L, Theeten H, Desmet S. Infant Pneumococcal Carriage in Belgium Not Affected by COVID-19 Containment Measures. Front Cell Infect Microbiol. 2021;11:825427. 35111700 10.3389/fcimb.2021.825427 PMC8801737

[24] Danino D, Ben-Shimol S, van der Beek BA, Givon-Lavi N, Avni YS, Greenberg D, Decline in Pneumococcal Disease in Young Children During the Coronavirus Disease 2019 (COVID-19) Pandemic in Israel Associated With Suppression of Seasonal Respiratory Viruses, Despite Persistent Pneumococcal Carriage: A Prospective Cohort Study. Clin Infect Dis. 2022;75:e1154–e64. 34904635 10.1093/cid/ciab1014 PMC8754767

[25] Loo JD, Conklin L, Fleming-Dutra KE, Knoll MD, Park DE, Kirk J, Systematic review of the indirect effect of pneumococcal conjugate vaccine dosing schedules on pneumococcal disease and colonization. Pediatr Infect Dis J. 2014;33 Suppl 2:S161–71. 24336058 10.1097/INF.0000000000000084 PMC3940524

[26] Kobayashi M, Bigogo G, Kim L, Mogeni OD, Conklin LM, Odoyo A, Impact of 10-Valent Pneumococcal Conjugate Vaccine Introduction on Pneumococcal Carriage and Antibiotic Susceptibility Patterns Among Children Aged <5 Years and Adults With Human Immunodeficiency Virus Infection: Kenya, 2009–2013. Clin Infect Dis. 2020;70:814–26. 30959526 10.1093/cid/ciz285 PMC6942635

[27] Nzenze SA, Madhi SA, Shiri T, Klugman KP, de Gouveia L, Moore DP, Imputing the Direct and Indirect Effectiveness of Childhood Pneumococcal Conjugate Vaccine Against Invasive Pneumococcal Disease by Surveying Temporal Changes in Nasopharyngeal Pneumococcal Colonization. Am J Epidemiol. 2017;186:435–44. 28482004 10.1093/aje/kwx048

[28] Roca A, Hill PC, Townend J, Egere U, Antonio M, Bojang A, Effects of community-wide vaccination with PCV-7 on pneumococcal nasopharyngeal carriage in the Gambia: a cluster-randomized trial. PLoS Med. 2011;8:e1001107. 22028630 10.1371/journal.pmed.1001107 PMC3196470

[29] Mackenzie GA, Hossain I, Salaudeen R, Badji H, Manjang A, Usuf E, Impact of pneumococcal conjugate vaccination on pneumococcal nasopharyngeal carriage in the Gambia: Population-based cross-sectional surveys. Vaccine. 2024;42:2680–6. 38490820 10.1016/j.vaccine.2024.02.066 PMC11004668

[30] Perdrizet J, Horn EK, Hayford K, Grant L, Barry R, Huang L, Historical Population-Level Impact of Infant 13-Valent Pneumococcal Conjugate Vaccine (PCV13) National Immunization Programs on Invasive Pneumococcal Disease in Australia, Canada, England and Wales, Israel, and the United States. Infect Dis Ther. 2023;12:1351–64. 37079175 10.1007/s40121-023-00798-x PMC10229489

[31] Verani JR, Omondi D, Odoyo A, Odiembo H, Ouma A, Ngambi J, Long-term impact of 10-valent pneumococcal conjugate vaccine in Kenya: Nasopharyngeal carriage among children in a rural and an urban site six years after introduction. Vaccine. 2024;42:126120. 39004525 10.1016/j.vaccine.2024.07.021 PMC11413479

[32] Dagan R, Patterson S, Juergens C, Greenberg D, Givon-Lavi N, Porat N, Comparative immunogenicity and efficacy of 13-valent and 7-valent pneumococcal conjugate vaccines in reducing nasopharyngeal colonization: a randomized double-blind trial. Clin Infect Dis. 2013;57:952–62. 23804191 10.1093/cid/cit428

[33] Andrews NJ, Waight PA, Burbidge P, Pearce E, Roalfe L, Zancolli M, Serotype-specific effectiveness and correlates of protection for the 13-valent pneumococcal conjugate vaccine: a postlicensure indirect cohort study. Lancet Infect Dis. 2014;14:839–46. 25042756 10.1016/S1473-3099(14)70822-9

[34] von Gottberg A, Kleynhans J, de Gouveia L, Tempia S, Meiring S, Quan V, Long-term effect of pneumococcal conjugate vaccines on invasive pneumococcal disease incidence among people of all ages from national, active, laboratory-based surveillance in South Africa, 2005–19: a cohort observational study. Lancet Glob Health. 2024;12:e1470–e84. 39151982 10.1016/S2214-109X(24)00263-8

